# Antimicrobial resistance in commensal opportunistic pathogens isolated from non-sterile sites can be an effective proxy for surveillance in bloodstream infections

**DOI:** 10.1038/s41598-021-02755-5

**Published:** 2021-12-03

**Authors:** Karina-Doris Vihta, Nicola Claire Gordon, Nicole Stoesser, T. Phuong Quan, Carina S. B. Tyrrell, Manivanh Vongsouvath, Elizabeth A. Ashley, Vilada Chansamouth, Paul Turner, Clare L. Ling, David W. Eyre, Nicholas J. White, Derrick Crook, Tim E. A. Peto, Ann Sarah Walker

**Affiliations:** 1 Nuffield Department of Clinical Medicine, University of Oxford, Oxford, UK; 2 National Institute for Health Research Health Protection Research Unit, Oxford, UK; 3 London School of Hygiene and Tropical Medicine, London, UK; 4 Oxford University Hospitals NHS Foundation Trust, Oxford, UK; 5 University of Cambridge, Cambridge, UK; 6 Lao-Oxford-Mahosot Hospital-Wellcome Trust Research Unit, Vientiane, Laos; 7 Centre for Tropical Medicine & Global Health, Nuffield Department of Medicine, University of Oxford, Oxford, UK; 8 Cambodia Oxford Medical Research Unit, Angkor Hospital for Children, Siem Reap, Cambodia; 9 Shoklo Malaria Research Unit, Mahidol-Oxford Tropical Medicine Research Unit, Faculty of Tropical Medicine, Mahidol University, Mae Sot, Thailand; 10 Big Data Institute, University of Oxford, Oxford, UK; 11 Mahidol Oxford Tropical Medicine Research Unit, Faculty of Tropical Medicine, Mahidol University, Bangkok, Thailand; 12 Microbiology Research Level 7, John Radcliffe Hospital, Headley Way, Oxford, OX3 9DU UK

**Keywords:** Epidemiology, Antimicrobials

## Abstract

Antimicrobial resistance (AMR) surveillance in bloodstream infections (BSIs) is challenging in low/middle-income countries (LMICs) given limited laboratory capacity. Other specimens are easier to collect and process and are more likely to be culture-positive. In 8102 *E. coli* BSIs, 322,087 *E. coli* urinary tract infections, 6952 *S. aureus* BSIs and 112,074 *S. aureus* non-sterile site cultures from Oxfordshire (1998–2018), and other (55,296 isolates) rarer commensal opportunistic pathogens, antibiotic resistance trends over time in blood were strongly associated with those in other specimens (maximum cross-correlation per drug 0.51–0.99). Resistance prevalence was congruent across drug-years for each species (276/312 (88%) species-drug-years with prevalence within ± 10% between blood/other isolates). Results were similar across multiple countries in high/middle/low income-settings in the independent ATLAS dataset (103,559 isolates, 2004–2017) and three further LMIC hospitals/programmes (6154 isolates, 2008–2019). AMR in commensal opportunistic pathogens cultured from BSIs is strongly associated with AMR in commensal opportunistic pathogens cultured from non-sterile sites over calendar time, suggesting the latter could be used as an effective proxy for AMR surveillance in BSIs.

## Introduction

Antimicrobial resistance (AMR) is among the top ten global health threats^1^, and is a particularly acute challenge in low- to middle-income countries (LMICs)^2–4^. Surveillance is a key tool to combat AMR, particularly in LMICs where lack of laboratory capacity prevents routine patient-level antimicrobial susceptibility testing^5^. The lack of surveillance data contributes to a pragmatic but broad-spectrum empiric treatment approach in hospitals with limited laboratory facilities^2,6^, generally resulting in overtreatment and acting as a selective pressure for AMR^7^. To date, surveillance capacity-building programmes have generally focused on implementing blood culture surveillance due to the high mortality of bloodstream infections (BSIs); however, blood cultures are a comparatively costly sample to process, require specialised laboratory equipment and trained staff due to the invasive nature of blood sampling, and can have slow turnaround times, reducing their utility for both clinical management and surveillance^8^. Additionally, the low positivity rate means that very high sample throughput is needed to confidently estimate AMR prevalence. Consequently, empirical treatment guidelines currently remain largely uninformed by AMR estimates from BSI surveillance in LMICs.

One solution to improve surveillance could be to assess AMR rates using data from non-invasive samples (e.g. urine, wound swabs), which are easier and faster to collect in large numbers, and easier to process in fledgling laboratories. Reflecting this, the 2020 WHO GLASS report includes AMR prevalence in urine cultures for key Gram-negative pathogens. However, associations between AMR rates in blood and other specimens have been studied in only a small number of longitudinal studies. Data from South Korea, the US and the UK have observed similar resistance rates in blood cultures and non-blood specimens (urine, faeces, skin/screening samples) for *Escherichia coli*, non-typhoidal *Salmonella enterica* and methicillin-resistant *Staphylococcus aureus*^9–12^. This raises the broader question as to whether, for multiple commensal opportunistic pathogen-drug combinations, the proportion of resistant BSIs could be estimated from the proportion of resistant isolates cultured from other body-sites consistently over calendar time (i.e. resistance trends), improving the power, feasibility and cost of culture-based AMR surveillance, and informing the development of locally empiric treatment guidelines^13^.

To evaluate this hypothesis, we first investigated the annual prevalence of and associations between AMR in commensal opportunistic pathogens isolated from BSIs and other body-sites for multiple pathogen-drug combinations in Oxfordshire [regional analysis, high-income country (HIC)]. We then extended the analysis, first to consider concurrent or previous isolates in the same patient, and second to consider the relevance of this approach in other world regions through publicly available datasets [Antimicrobial Testing Leadership and Surveillance (ATLAS)^14^] and collaborating hospitals/programmes in LMICs.

## Results

### AMR prevalence across antimicrobial classes in *E. coli* UTIs is strongly associated with AMR prevalence in *E. coli* BSIs in Oxfordshire, UK

**Figure 1:**
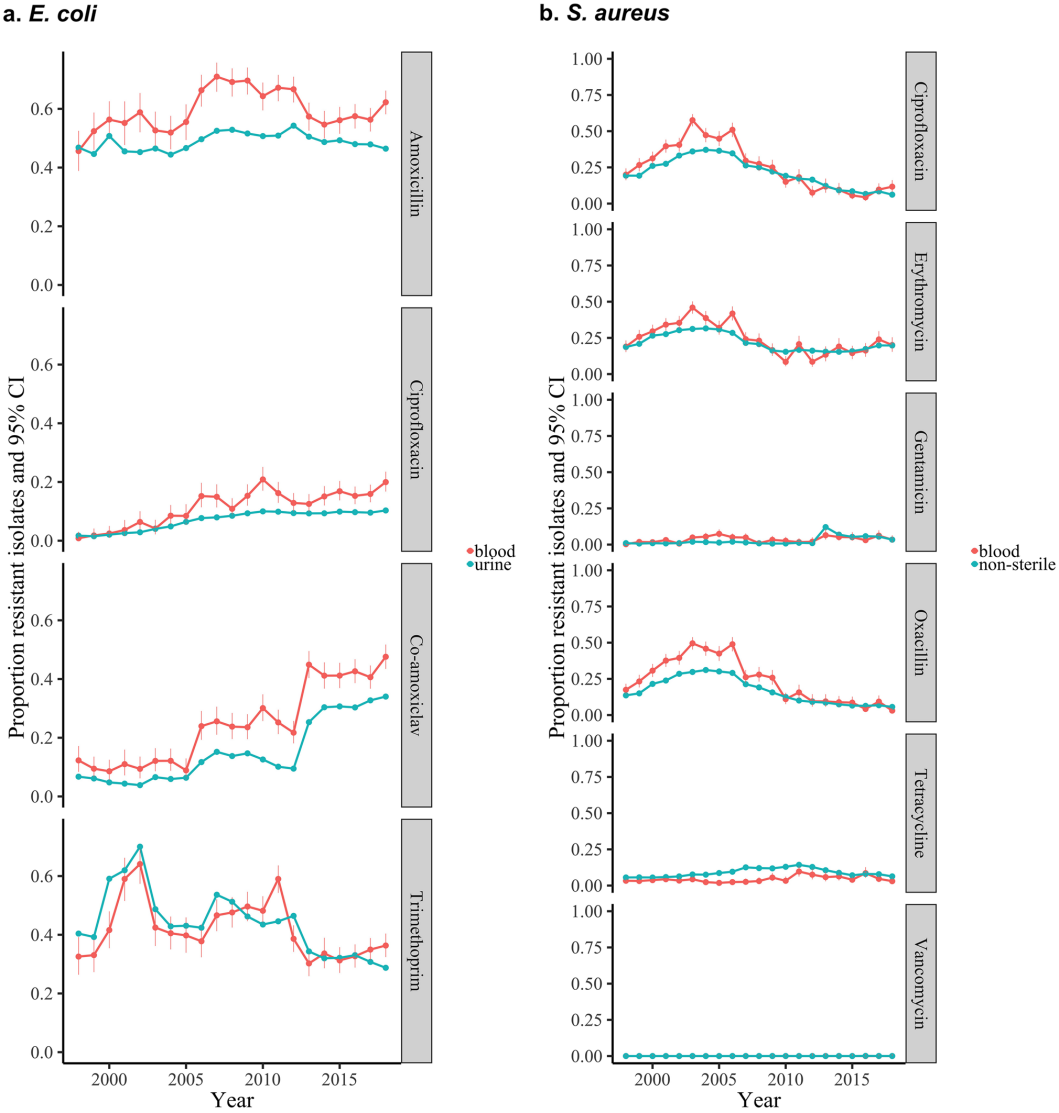
Resistance prevalence in *E. coli* and *S. aureus* in blood versus non-blood cultures in Oxfordshire, UK, 1998–2018 maximum cross-correlation at time lag 0 in 3/4 drugs (ciprofloxacin, co-amoxiclav, trimethoprim) for *E. coli* and 4/6 drugs for *S. aureus* (ciprofloxacin, erythromycin, gentamicin, oxacillin); cross-correlation 0.77 at lag 1 (0.75 at lag 0), 0.95 at lag 0, 0.96 at lag 0, 0.80 at lag 0 for *E. coli* and 0.95 at lag 0, 0.93 at lag 0, 0.54 at lag 0, 0.75 at lag 0, 0.69 at lag − 4, 0.60 at lag 3 for *S. aureus* from top to bottom respectively (full results in Supplementary Table S1).

We focused initially on *E. coli* as the most common bacteria causing BSIs and UTIs^2,15,16^. We included 8,102 *E. coli* BSIs and 322,087 *E. coli* UTIs (40-fold higher) from Oxfordshire (Supplementary Fig. S1); four antibiotics (amoxicillin, co-amoxiclav, ciprofloxacin, and trimethoprim) were consistently tested in both between 1998 and 2018. Whilst absolute resistance prevalence was generally slightly higher in bloodstream isolates, trends in the proportion of resistant BSIs and UTIs were very similar, notably tracking substantial fluctuations over time in trimethoprim resistance (Fig. 1, Supplementary Fig. S2, Supplementary Table S1), with resistance rates in the same year most strongly correlated (i.e. maximum cross-correlation at lag 0; Supplementary Table S1). For these four antibiotics, there was no evidence of strong variation in the relationship between resistance rates over time.

**Figure 2:**
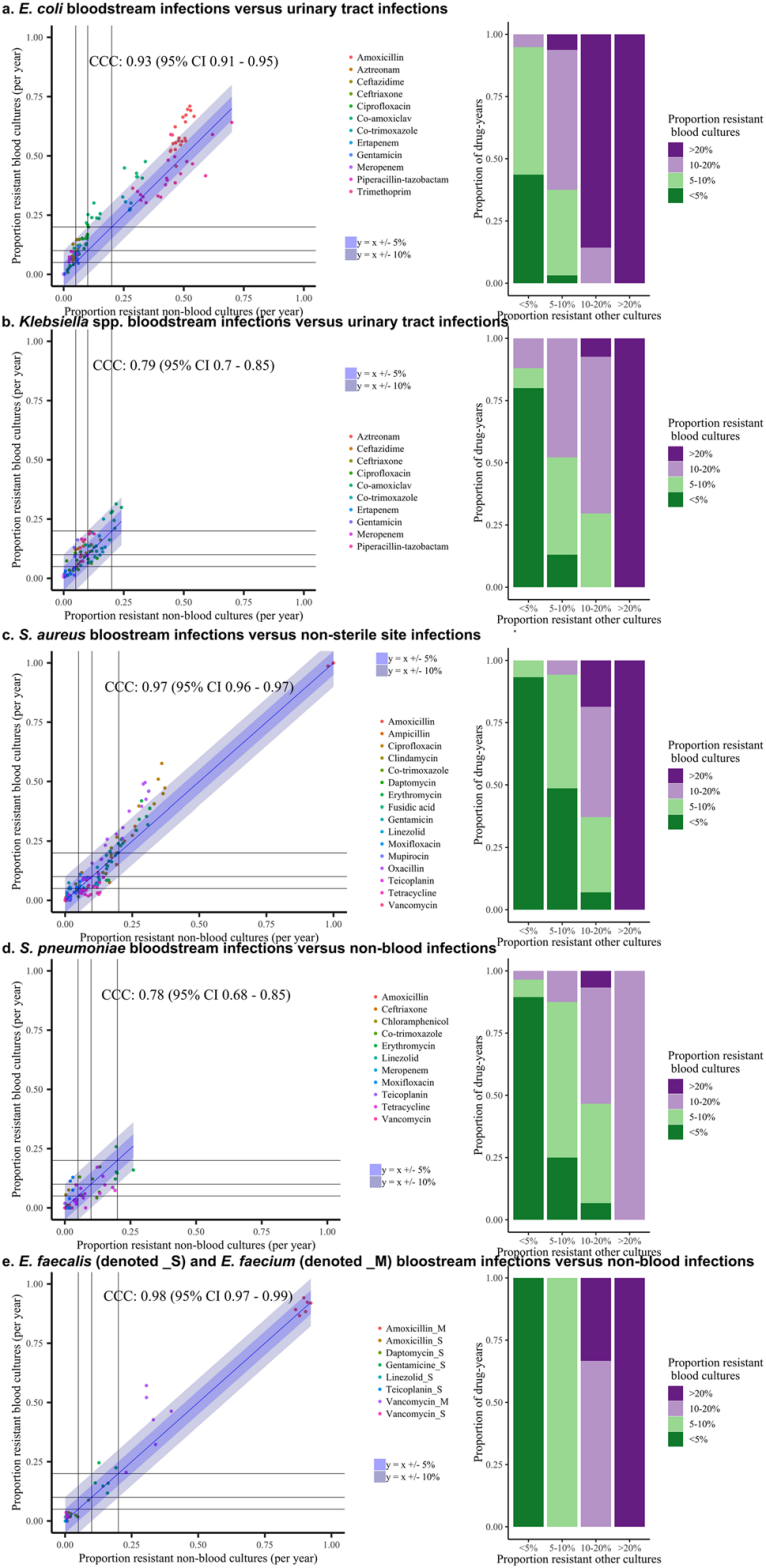
Association between resistance prevalence in blood versus non-blood cultures for (**a**) *E. coli*, (**b**) *Klebsiella *spp., (**c**) *S. aureus*, (**d**) *S. pneumoniae*, (**e**) *E. faecalis* and *E. faecium*, Oxfordshire 1998–2018 Left: Scatterplot of the proportion of resistant non-blood cultures versus the proportion of resistant blood cultures per year for all antibiotics. Each dot reflects resistance prevalence for one antibiotic in one specific year, coloured by antibiotic type. Line of identity shown in blue, with associated ± 5% and ± 10% agreement discordance intervals. Additional lines are drawn to denote 0.05 (5%), 0.1 (10%) and 0.2 (20%) resistance prevalence thresholds for blood (horizontal lines) versus non-blood (vertical lines) cultures for ease of visualisation of agreement between clinically meaningful resistance categories in these two types of samples. Right: Stacked bars representing the data in the left-hand panel grouped as categorical resistance prevalence ranges, classifying resistance prevalence in blood cultures by the proportion in urine/non-sterile site (i.e. non-blood) cultures.

For a further eight antibiotics (aztreonam, ceftazidime, ceftriaxone, co-trimoxazole, ertapenem, gentamicin, meropenem and piperacillin-tazobactam) consistently tested between 2013 and 2018, again, resistance rates were slightly higher in BSIs and showed reasonable concordance with resistance in UTIs (Supplementary Fig. S3, Supplementary Tables S1, S2), with prevalences within 10% of each other in 109/132 (89%) drug-years (Fig. 2, left-hand panel, Table 1). Overall for *E. coli*, resistance prevalence in BSIs was highly correlated with resistance prevalence in UTI (CCC = 0.93 (95% CI 0.91–0.95) (Table 1). Agreement was also relatively high across our four pre-defined resistance prevalence categories which might affect empirical prescribing practice, with 83/132 (63%) drug-years in the same prevalence category and 45/132 (34%) in adjacent prevalence categories (Fig. 2, right-hand panel).

**Table 1:** Resistance prevalence in blood and non-blood cultures across drug-years, Oxfordshire 1998–2018.

Pathogen	Lin’s concordance coefficient (95% CI)	< 5% difference in resistance prevalence	< 10% difference in resistance prevalence	Same resistance prevalence category^a^	One resistance prevalence category^a^ different	Two resistance prevalence categories^a^ different
**Oxfordshire**						
*E. coli*	0.93 (0.91–0.95)	67/132 (51%)	109/132 (83%)	83/132 (63%)	45/132 (34%)	4/132 (3%)
*Klebsiella* spp*.*	0.79 (0.69–0.85)	54/80 (68%)	79/80 (99%)	51/80 (64%)	26/80 (32%)	3/80 (4%)
*S. aureus*	0.97 (0.95–0.97)	140/180 (78%)	167/180 (93%)	132/180 (73%)	45/180 (25%)	3/180 (2%)
*S. pneumoniae*	0.78 (0.68–0.85)	63/81 (78%)	79/81 (98%)	63/81 (78%)	15/81 (19%)	3/81 (4%)
*E. faecalis*	0.90 (0.82–0.94)	35/36 (97%)	35/36 (97%)	34/36 (94%)	2/36 (6%)	0/36 (0%)
*E. faecium*	0.92 (0.77–0.97)	8/12 (67%)	9/12 (75%)	12/12 (100%)	0/12 (0%)	0/12 (0%)
**ATLAS high-income countries**						
*E. coli*	0.97 (0.96–0.97)	302/401 (75%)	376/401 (94%)	326/401 (81%)	74/401 (18%)	1/401 (0%)
*S. aureus*	0.99 (0.99–0.99)	140/155 (90%)	147/155 (95%)	154/155 (99%)	1/155 (1%)	0/155 (0%)
*K. pneumoniae*	0.89 (0.86–0.92)	135/186 (73%)	172/186 (92%)	134/186 (72%)	51/186 (27%)	1/186 (1%)
*P. aeruginosa*	0.89 (0.80–0.94)	27/35 (77%)	34/35 (97%)	25/35 (71%)	10/35 (29%)	0/35 (0%)
**ATLAS middle-income countries**						
*E. coli*	0.95 (0.94–0.96)	157/268 (59%)	213/268 (79%)	234/268 (87%)	31/268 (12%)	3/268 (1%)
*S. aureus*	0.96 (0.95–0.97)	260/344 (76%)	308/344 (90%)	289/344 (84%)	47/344 (14%)	8/344 (2%)
*K. pneumoniae*	0.86 (0.82–0.89)	87/228 (38%)	146/228 (64%)	170/228 (75%)	53/228 (23%)	5/228 (2%)
*P. aeruginosa*	0.77 (0.60–0.88)	20/36 (56%)	31/36 (86%)	32/36 (89%)	4/36 (11%)	0/36 (0%)
*E. cloacae*	0.96 (0.93–0.97)	31/61 (51%)	45/61 (74%)	49/61 (80%)	10/61 (16%)	2/61 (3%)
**Cambodia**						
*E. coli*	0.67 (0.53–0.78)	16/70 (23%)	27/70 (39%)	63/70 (90%)	4/70 (6%)	3/70 (4%)
*S. aureus*	0.97 (0.95–0.98)	22/48 (46%)	39/48 (81%)	30/48 (62%)	15/48 (31%)	3/48 (6%)
**Laos**						
*E. coli*	0.83 (0.64–0.92)	3/20 (15%)	10/20 (50%)	20/20 (100%)	0/20 (0%)	0/20 (0%)
*S. aureus*	0.96 (0.85–0.99)	4/10 (40%)	8/10 (80%)	7/10 (70%)	3/10 (30%)	0/10 (0%)
**Thailand**						
*E. coli*	0.91 (0.87–0.94)	48/120 (40%)	77/120 (64%)	77/120 (64%)	34/120 (28%)	9/120 (8%)
*S. aureus*	0.97 (0.96–0.98)	56/84 (67%)	72/84 (86%)	55/84 (65%)	21/84 (25%)	8/84 (10%)

^a^Resistance prevalence categories are: < 5%, 5–10%, 10–20%, > 20%.

In the Oxfordshire dataset, non-blood cultures are urine for *E. coli* and *Klebsiella* spp., other non-sterile sites for *S. aureus* and all other body sites for other Gram-negatives and *S. pneumoniae*, *E. faecalis* and *E. faecium*; in the ATLAS dataset non-blood cultures are all other body (i.e. non-blood) sites for all pathogens; in the three hospitals from LMICs non-blood cultures are urine for *E. coli* and swabs/pus for *S. aureus.*

Considering matched pairs of isolates from individual patients, there was no evidence that the modestly different resistance rates seen in *E. coli* BSIs and UTIs overall were due to differential sampling of intrinsically different underlying populations, given high but similarly imperfect agreement in susceptibility (86–100% across 11 antibiotics for concurrent infections, 80–94% for previous infections, Table S3). In particular, considering the closest prior *E. coli* UTI 3–90 days before a BSI, > 80% UTIs resistant to amoxicillin, co-amoxiclav, ciprofloxacin, and trimethoprim shared the same resistance phenotype in the subsequent BSI.

### AMR prevalence across antimicrobial classes in *S. aureus* from sterile and non-sterile sites is strongly associated with AMR prevalence in *S. aureus* BSIs in Oxfordshire, UK

Next we considered similar analyses for *S. aureus*, as one of the commonest causes of Gram-positive BSI and skin/soft tissue infections. Between 1998 and 2018, there were 6952 *S. aureus* BSIs and 166,179 *S. aureus* non-blood cultures in Oxfordshire: 54,105 (33%) from sterile sites and 112,074 (67%) from non-sterile sites (8- and 16-fold higher respectively, Supplementary Fig. S1). Resistance trends for ciprofloxacin, erythromycin and oxacillin were similar for the different sample types, and varied substantially over time (Fig. 1, Supplementary Table S1, Supplementary Fig. S4), again supporting the possibility of using resistance prevalence in non-blood cultures as a proxy for resistance prevalence in blood cultures. Agreement in resistance prevalence between blood/non-blood samples was poorer for gentamicin and tetracycline resistance, although resistance rates were low (< 15%). As might be expected, given the propensity of *S. aureus* to cause purulent metastatic infection in association with BSI, agreement was slightly better between resistance prevalence in blood and other sterile site cultures than blood and non-sterile site cultures (Supplementary Fig. S5).

As non-sterile site cultures are easier to collect and would be more likely to be taken in settings with limited healthcare capacity, and agreement with resistance prevalence in BSIs was reasonable, we focussed on this group of isolates. Overall, resistance prevalence was more similar in bloodstream and other non-sterile infection sites in *S. aureus* than *E. coli* (Supplementary Tables S1, S2), but there were also slightly stronger effects of calendar time for *S. aureus*, reflecting generally higher rates of resistance in blood compared with non-sterile site cultures during the MRSA epidemic in the mid-2000s, which subsequently became more similar (Supplementary Fig. S3).

For *S. aureus*, including nine other antibiotics (amoxicillin, clindamycin, co-trimoxazole, daptomycin, fusidic acid, linezolid, moxifloxacin, mupirocin, teicoplanin) tested between 2013 and 2018 only, resistance prevalences in blood and non-sterile site cultures were within 10% of each other in 167/180 (93%) drug-years, with 132 (73%) drug-years in the same resistance category and 45 (25%) in adjacent categories (Fig. 2, Table 1). Overall resistance prevalence in blood was highly correlated with resistance prevalence in non-sterile isolates [CCC = 0.97 (0.95–0.97)] (Table 1).

Within individual patients, overall agreement between resistance in concurrent blood and non-sterile cultures was extremely high (96–100%) for *S. aureus*, with ciprofloxacin, erythromycin and oxacillin having < 5% of resistant blood cultures with susceptible non-sterile cultures and < 4% susceptible blood cultures with resistant non-sterile sites cultures (Supplementary Table S3).

### AMR prevalence across antimicrobial classes in *Klebsiella* spp., *Streptococcus pneumoniae* and *Enterococcus* spp. from blood and non-blood specimens is also strongly associated in Oxfordshire, UK

Although numbers were smaller than for *E. coli* and *S. aureus*, broadly similar associations in resistance prevalence between blood/non-blood isolates were seen for *Klebsiella* spp. (2536 BSIs and 24,192 non-blood cultures) (Fig. 2, Table 1, Supplementary Figs. S6, S7, Supplementary Tables S1, S2), and several additional common Gram-positive pathogens, including *S. pneumoniae* (1703 BSIs and 7369 non-blood cultures), *E. faecalis* (1218 BSIs and 15,317 non-blood cultures) and *E. faecium* (934 BSIs and 2027 non-blood cultures) (Fig. 2, Table 1, Supplementary Fig. S8, Supplementary Tables S1, S2). For *Klebsiella* spp. resistance prevalences in blood and non-blood isolates were within 10% of each other in 79/80 (99%) drug-years, with 51 (64%) drug-years in the same resistance category and 26 (32%) in adjacent categories (Fig. 2, Table 1); for *S. pneumoniae* 79/81 (98%), 63 (78%), 15 (19%), respectively; for *E. faecalis* 35/36 (97%), 34 (94%), 2 (6%), respectively; for *E. faecium* 9/12 (75%) and with all 12 drug-years in the same resistance category.

### AMR prevalence in blood and non-blood sites across antimicrobial classes and common pathogens is also strongly associated within 17 countries in the ATLAS dataset

**Figure 3:**
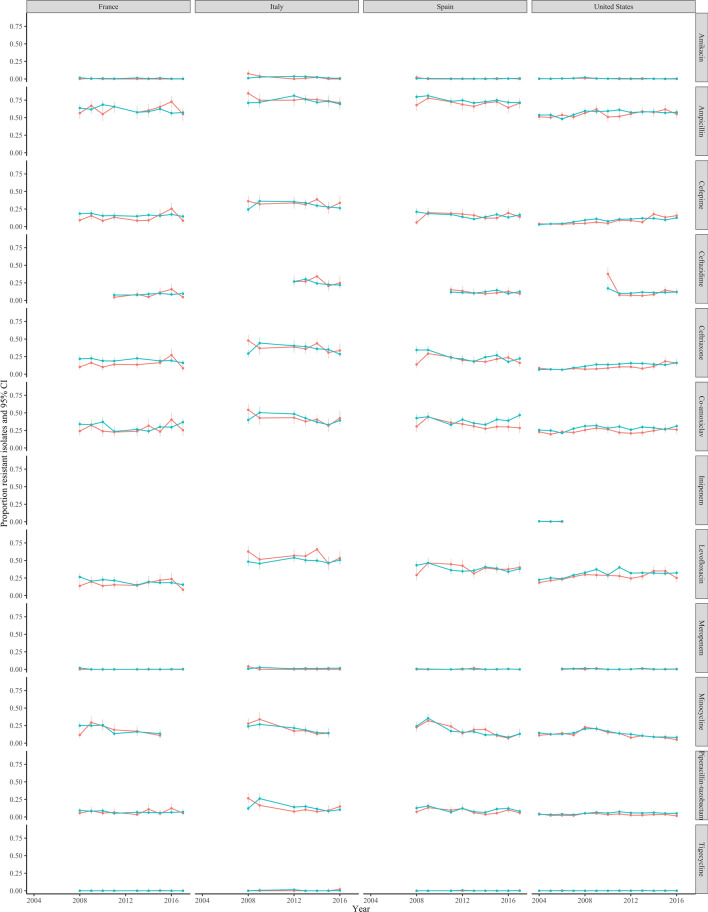
Resistance prevalence in *E. coli* in blood versus non-blood cultures in high-income countries present in the ATLAS dataset, 2004–2016.

**Figure 4:**
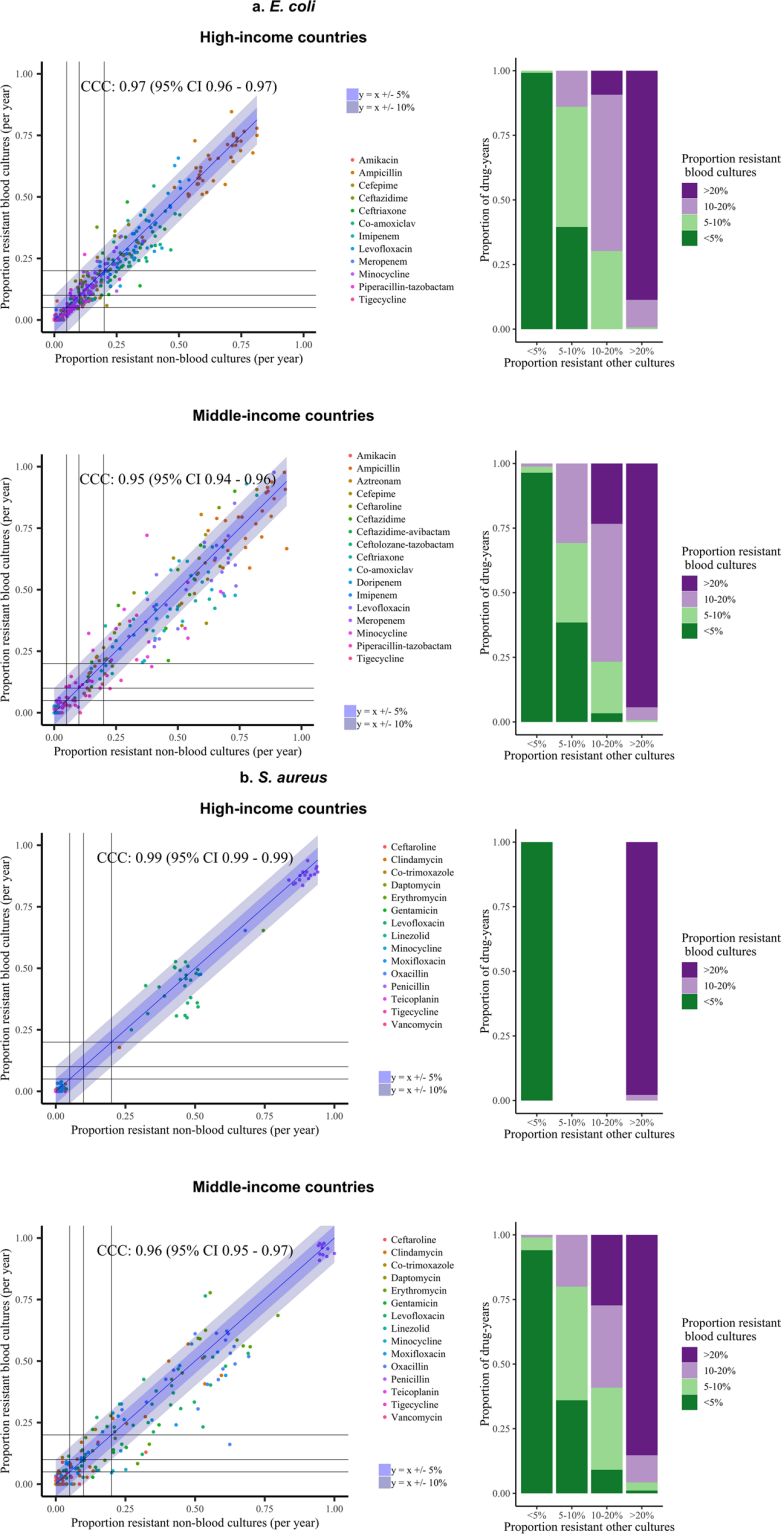
Association between resistance prevalence in blood versus non-blood cultures for: (**a**) *E. coli*, (**b**) *S. aureus*, in the ATLAS dataset, split into high-income countries and middle-income countries. Left: Scatterplot of the proportion of resistant non-blood cultures versus the proportion of resistant blood cultures per year for all antibiotics. Each dot reflects resistance prevalence for one antibiotic in one specific year, coloured by antibiotic type. Line of identity shown in blue, with associated ± 5% and ± 10% agreement discordance intervals. Additional lines are drawn to denote 0.05 (5%), 0.1 (10%) and 0.2 (20%) resistance prevalence thresholds for blood (horizontal lines) versus non-blood (vertical lines) cultures for ease of visualisation of agreement between clinically meaningful resistance categories in these two types of samples. Right: Stacked bars representing the data in the left-hand panel grouped as categorical resistance prevalence ranges, classifying resistance prevalence in blood cultures by the proportion in urine/non-sterile site (i.e. non-blood) cultures.

In the ATLAS dataset, there were 401 and 155 country-drug-years with > 100 *E. coli* and *S. aureus* isolates from HICs, respectively, from both bloodstream and urinary/non-sterile infections (Supplementary Table S4). For *E. coli*, time trends could be estimated over 7 years or more in four countries for 11 antibiotics, and were broadly similar (Fig. 3; *S. aureus* in Supplementary Fig. S9). Across all drugs, prevalence of resistance in *E. coli* BSIs and UTIs were within 10% of each other in 376/401 (94%) country-drug-years (Fig. 4, left-hand panel, Table 1); 326 (81%) country-drug-years were in the same resistance category and 74 (18%) in adjacent categories (Fig. 4, right column). For *S. aureus*, respective figures were 147/155 (95%), 154 (99%) and 1 (1%). Agreement was similarly high in middle-income countries where smaller numbers of isolates were available (Fig. 4, Table 1, Supplementary Figs. S10, S11), for *E. coli* 213/268 (80%), 234 (87%), 31 (12%), respectively, for *S. aureus* 308/344 (90%), 289 (84%), 47 (14%), respectively; and also in other pathogens (Table 1, Supplementary Figs. S12–S16), for *K. pneumoniae* 146/228 (64%), 170 (75%), 53 (23%), respectively, for *P. aeruginosa* 31/36 (86%), 32 (89%), 4 (11%), respectively, and for *E. cloacae* 45/61 (74%), 49 (80%), 10 (16%) respectively,.

### AMR prevalence in blood and non-blood sites across antimicrobial classes and common commensal opportunistic pathogens is also associated within three LMICs

**Figure 5:**
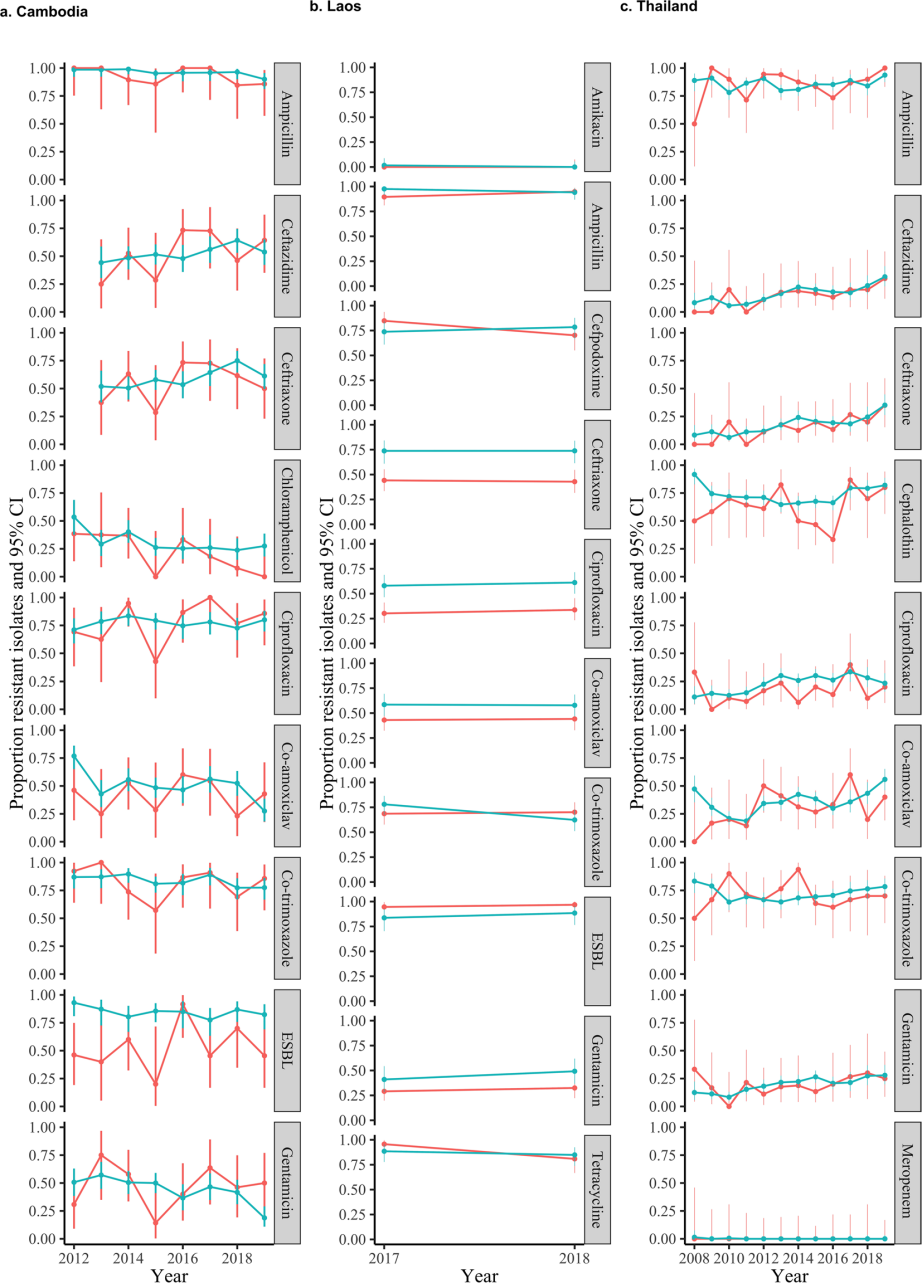
Resistance prevalence in *E. coli* in blood versus non-blood cultures in three hospitals/programmes in LMICs.

**Figure 6:**
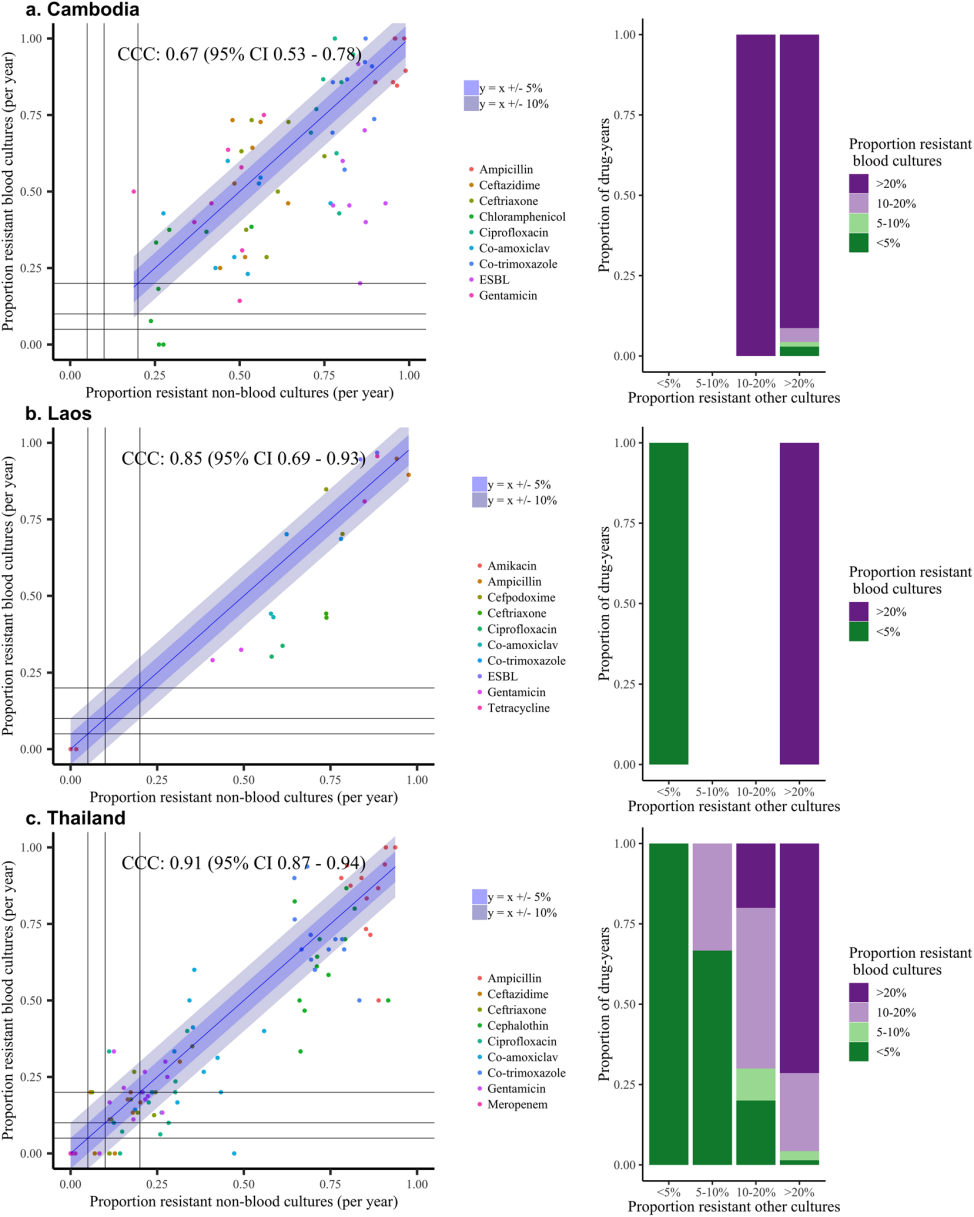
Association between resistance prevalence in *E. coli* blood versus urine cultures in three hospitals/programmes in LMICs. Left: Scatterplot of the proportion of resistant non-blood cultures versus the proportion of resistant blood cultures per year for all antibiotics. Each dot reflects resistance prevalence for one antibiotic in one specific year, coloured by antibiotic type, with 21 dots per antibiotic (one for each year 1998–2018). Line of identity shown in blue, with associated ± 5% and ± 10% agreement discordance intervals. Additional lines are drawn to denote 0.05 (5%), 0.1 (10%) and 0.2 (20%) resistance prevalence thresholds for blood (horizontal lines) versus non-blood (vertical lines) cultures for ease of visualisation of agreement between clinically meaningful resistance categories in these two types of samples. Right: Stacked bars representing the data in the left-hand panel grouped as categorical resistance prevalence ranges, classifying resistance prevalence in blood cultures by the proportion in urine/non-sterile site (i.e. non-blood) cultures*.*

Considering hospital/programme-level LMIC datasets, numbers were smaller with 6154 isolates in total only (Supplementary Table S5 contains the minimum and maximum number of blood and non-blood cultures per year per pathogen for each dataset, minimum 5, maximum 327). Estimates were therefore more variable, but both time trends (Fig. 5, Supplementary Fig. S17) and comparisons of individual country-drug-years (Fig. 6, Table 1, Supplementary Fig. S18) supported overall findings that resistance profiles in non-blood culture isolates correlate well with those in blood culture isolates over time. For the datasets from Cambodia, Laos and Thailand, *E. coli* resistance prevalences in blood and non-blood isolates were within 10% of each other in 27/70 (39%), 10/20 (50%) and 77/120 (64%) drug-years respectively, with 63/70 (90%), 20/20 (100%), 77/120 (64%) drug-years respectively in the same resistance category and 4/70 (6%), 0/20 (0%), 34/120 (28%) in adjacent categories (Fig. 6, Table 1). For *S. aureus*, for Cambodia respective figures were 39/48 (81%), 30 (62%), 15 (31%), for Laos 8/10 (80%), 7 (70%), 3 (30%), and for Thailand 72/84 (86%), 55 (65%) and 21 (25%) respectively.

## Discussion

Here, we show that across countries and World Bank income status (i.e. high, middle, low income status), AMR prevalence in commensal opportunistic pathogens cultured from non-blood samples could potentially be used as a proxy for AMR prevalence in BSIs within a given setting. Focusing on two of the most common bacteria causing serious infections, *E. coli* and *S. aureus*, we found that, whilst not perfect, agreement between AMR rates in blood cultures and in other clinical cultures was good for most antibiotics, and, importantly, consistently associated over time. Similar results were observed for *Klebsiella *spp., *S. pneumoniae*, *E. faecalis*, *E. faecium, P. aeruginosa* and *E. cloacae*. This could be of particular relevance to reducing the costs and logistics of AMR surveillance in LMICs, and improving the speed with which AMR prevalence data can be generated, given that urine and skin swab samples are easy to collect and more straightforward to culture than blood, with higher positivity rates. Reliable local AMR prevalence data could then be used to better inform the appropriateness of different empirical antimicrobial guidelines.

Whilst many studies estimate resistance prevalence in various infection sites, to our knowledge, very few studies have directly compared resistance in blood versus non-blood sites, and these essentially focus only specific drug-pathogen combinations^9,11,12^. In 2011, Health Protection Scotland and National Services Scotland published a protocol for the surveillance of AMR in UTIs, arguing that it would be more relevant and feasible to monitor emerging multidrug resistant strains and changes in proportions of resistance in UTIs than in BSIs^17^. However, the hypothesised relationship was not analysed using the data collected^18^. The WHO piloted a similar approach but did not compare with resistance rates in BSIs^19^. The use of metagenomics on pooled colonisation samples as a proxy measure for AMR prevalence in populations has also been proposed as a strategy for rapidly generating reliable AMR prevalence estimates to inform prescribing guidelines^20^.

Current efforts on AMR surveillance in LMICs are directed towards building laboratories and training staff to carry out antimicrobial susceptibility testing on blood cultures, arguing that if this can be achieved, other sample cultures will become possible too. However, setting up blood culture processing is very expensive, both in terms of infrastructural and running costs, and because the microbiological yield from blood cultures is low, it will be many years until sufficient data on AMR prevalence can be accumulated for surveillance purposes. Incorporating blood culture testing in public hospitals may also be significantly biased by differences in the ability of patients to pay^2^. Longitudinal surveillance data on AMR prevalence in BSIs is available at a limited number of research sites, but these programmes cover a limited geographic area and may not be representative of other locations in a country.

Collecting specimens from urine/skin surface/genital sites is cheaper and less invasive than sampling blood, and therefore substantially easier to do on large numbers from different communities, as well as having higher positivity rates, increasing the utility of microbiological sampling using these sample types for AMR surveillance. This is true even in HICs, as seen in this study, with much narrower confidence intervals around AMR prevalence estimates derived from much larger numbers of non-blood Oxfordshire isolates. Even allowing for the greater manual processing needed for non-blood cultures, yield in terms of resistance antibiograms is almost certainly far greater from the same number of non-sterile specimens compared to blood specimens. Whilst the specific positivity rates and proportion of pathogens from each species will vary across settings, as an example based on Oxfordshire data 1998–2016, obtaining 100 *E. coli* isolates would only require the testing of 361 urine samples (assuming 37% are culture-positive and 75% of these are *E. coli*), but 7693 blood cultures (assuming 13% are positive and 10% of these are *E. coli*.

A major strength of our study is the different datasets used and the different commensal opportunistic pathogens studied (with varying AMR prevalence), and the robustness of findings across these, including detailed, continuous surveillance of one large region (Oxfordshire), a global analysis across 17 countries (ATLAS), and specific evaluations in three LMICs—although the datasets in these three countries were relatively small. A crucial underpinning assumption is that pathogens causing non-bloodstream infections are representative of pathogens causing BSI in terms of antibiogram, either because the source of infection is commonly from commensal colonising organisms that become pathogenic opportunistically or because both types of infection arise from a similar reservoir; this assumption seems reasonable. If the former is more common, then one might hypothesise that this would occur more often from resistant colonising organisms as these are more likely to be unsuccessfully treated with antibiotics in the community, potentially explaining the generally slightly higher resistance in bloodstream compared to non-bloodstream specimens. One limitation of our study is that we only had enough numbers to analyse the relationship for eight commensal opportunistic pathogens. Our results may therefore not generalise to all other pathogens, although there was no indication that this would be the case from the ones that we were able to include, and, by definition, the included pathogens are the most common causing infections. An important limitation of our approach could be that different population sub-groups were sampled for blood and other specimens; however, our analysis of blood/non-blood samples from the same patients suggests this is unlikely to cause major bias. We have also not considered AMR surveillance encompassing phenotypic profiles (i.e. patterns of resistance within one isolate across different antibiotics), as opposed to discrete pathogen-drug combinations. We also pooled all samples together: future work could consider whether relationships are generalizable between different population sub-groups (e.g. by age, nosocomial/community infections). Finally, the resistance prevalence categories we considered were slightly arbitrary, which is why we considered two different approaches (varying margins of error and varying categorical thresholds).

Although different absolute resistance rates were observed in blood versus non-blood samples for many pathogen-drug combinations, cross-correlations were generally high, as was agreement within 10% and across AMR prevalence categories that might affect empiric antimicrobial prescribing practice. Several biological reasons for the lack of perfect agreement are plausible: for example, a large proportion of *E. coli* BSIs have a urinary focus, but appropriate empiric treatment generally limits progression to BSI, in contrast to resistant, and perhaps sub-optimally treated UTIs. Hence one might expect the proportion of drug-resistant BSIs to be higher, but underlying time trends to track each other, as we observed. Performance did vary slightly between different pathogen-drug combinations as well as between different locations. However, the totality of evidence across all the datasets supports the value of using non-sterile site specimens for antimicrobial surveillance, regardless of the income of the country.

In HICs, susceptibility testing still typically takes at least 48 h from sampling to result, during which empiric antimicrobials are administered, potentially leading to poorer outcomes if the infection is caused by a resistant organism^4^. However, increasing use of electronic health records means it is becoming much simpler to check for previous UTIs and use this to guide empiric treatment at the patient-level. Our paired analysis illustrates the potential of this approach based on an arbitrarily chosen interval of 3–90 days prior to the BSI; future work could investigate how the strength of this association varies further back in time, or using the most resistant rather than most recent urinary isolate. Similarly, Yelin et al*.*^21^ found that incorporating demographic information with susceptibility results of previous UTIs and antibiotic purchases in a machine learning model improved guidance of empirical treatment for new community-acquired UTIs.

AMR surveillance plays a key role in optimising the use of antibiotics, developing empiric treatment guidelines, and improving antimicrobial stewardship. Lack of microbiology facilities and unwillingness of patients to have samples taken (often due to cost) have been identified as key factors influencing antibiotic use^22,23^. LMICs face numerous challenges in setting up surveillance systems similar to those in HICs, including lack of coherent governance, budget, technical expertise, information technology systems and co-ordination. Although capacity-building and optimising diagnostic infrastructures in LMICs is clearly important, our study highlights that a bridging strategy using body-sites which are easier to sample, cheaper, faster and easier to culture compared to blood could provide a more rapidly scalable approach to AMR surveillance, providing evidence for empiric treatment recommendations. This sampling strategy could also be amenable to intermittent population survey-type approaches as opposed to continuous surveillance. Using population-level surveillance as a stepping-stone complementary to developing adequate infrastructure to support individual patient-level testing could be considered a pragmatic first step to informing AMR prevalence estimates and empiric treatment guidelines in resource-limited settings.

## Materials and methods

### Experimental design

#### Oxfordshire, UK, dataset (regional analysis, high-income country)

We obtained antibiograms (i.e. phenotypic profiles) for *E. coli, Klebsiella* spp., *Staphylococcus aureus, Streptococcus pneumoniae, Enterococcus faecalis* and *Enterococcus faecium* from the Infections in Oxfordshire Research Database (IORD)^24^, from 1 January 1998 to 31 December 2018. IORD includes all microbiology tests performed in Oxfordshire, UK, with a population size of ~ 680,000 individuals, and has Research Ethics Committee and Confidentiality Advisory Group approval (19/SC/0403, 19/CAG/0144) as a de-identified electronic research database. We compared *E. coli* and *Klebsiella* cultured from BSIs versus urinary tract infections (UTI); and isolates from BSIs versus all other body-sites for other pathogens (i.e. non-BSIs; excluding surveillance swabs), further sub-divided into sterile versus non-sterile samples for *S. aureus* (Supplementary Methods); total 558,616 isolates. We estimated yearly resistance prevalence for all antimicrobials with susceptibility data for > 65% samples in that year (mostly > 80%). In Oxfordshire, testing used manual disk diffusion before 2013, and thereafter automated testing with the BD Phoenix™ Automated Microbiology System (Becton Dickinson, NJ, USA). Binary susceptibility (i.e. susceptible/resistant) phenotypes were defined following laboratory standard operating procedures in place at the time of any given test, and using relevant European Committee on Antimicrobial Susceptibility testing (EUCAST) breakpoints for all specimen types^25^, dropping a very small number of intermediate isolates. We did not de-duplicate isolates by patient, in order to reflect the data that would be easily available from routine laboratory systems.

### Statistical analysis

For each pathogen-drug combination within the Oxfordshire dataset, we first estimated the proportion resistant in blood versus other samples in each calendar year (with 95% confidence intervals (CI)) using Lin’s concordance correlation coefficient (CCC) for comparisons^26^. We used time-series cross-correlation functions to identify the time interval at which the correlation between resistance prevalence was strongest. As perfect agreement is unrealistic, we also considered agreement within what might be considered clinically acceptable error by infectious disease specialists (± 5% and ± 10% difference in prevalence). In addition, for each pathogen-drug combination, we considered four arbitrary categories of AMR prevalence which might affect empirical prescribing practice, specifically: < 5% resistance prevalence (most prescribers would readily prescribe the antibiotic empirically), 5–10% resistance prevalence (most would prescribe for mild infections), 10–20% resistance prevalence (prescribers might consider alternative antibiotics as first-line empiric choices) and > 20% resistance prevalence (many prescribers would not prescribe if alternative options were available). These thresholds are arbitrary and so we also present exact estimates in Figures to demonstrate robustness to their choice. We used logistic random-effects meta-analysis to estimate the overall difference between resistance prevalence in the two sample types (i.e. blood versus non-blood), treating every calendar year as an independent study, assessing heterogeneity across years using the I^2^ statistic^27^. We used meta-regression to estimate the effect of calendar year on the proportion of resistant bloodstream isolates (with its standard error), assuming the proportion of resistant isolates from other infection sites was known, given their greater numbers. We also used meta-regression to directly estimate the association between log odds of resistance in bloodstream infections (outcome, with its standard error) and the log odds of resistance in other infection sites (explanatory variable, fixed). (See Supplementary Material for full details).

Resistance rates could differ between blood and other infection sites because of differences in the underlying populations being sampled. A secondary analysis therefore considered matched pairs of isolates from the same patient, selecting the closest culture from a different site up to 3 days before or 2 days after the blood culture^11^, assessing concordance using McNemar’s test. We also considered whether susceptibility of a previous *E. coli* UTI could predict susceptibility of a subsequent *E. coli* BSI, by selecting the temporally closest urine culture taken between 3 and 90 days before the blood culture^11,28^ and considering major/very major error rates of predictions^29^. While the time intervals are arbitrary, they have been used in the literature before and have been guided by the standard definition of a recurrent UTI episode^11,28^.

#### Antimicrobial Testing Leadership and Surveillance (ATLAS) dataset (country-level analyses, low/middle/high-income countries)

We also analysed pathogen-drug combinations at the country level (pooling multiple hospitals/regions) using the ATLAS dataset, comprising 103,559 isolates from 17 countries between 2004 and 2017^14^. Intermediate isolates were considered non-susceptible. We considered all years where at least 30 isolates were tested for a given drug in LMICs (95% CI width around prevalence always < 37%); and 100 isolates for HIC (width < 20%) given greater numbers from HIC and strong inverse association between numbers tested and resistance prevalence in HIC, suggesting preferential testing of resistant isolates. Resistance proportions, time-series cross-correlations, extent of agreement, and meta-analyses/regression were performed as for the Oxfordshire dataset above.

#### Cambodia, Laos, Thailand datasets (local level analyses, low-middle income countries)

Finally, we also analysed microbiology data through collaborative programmes at Angkor Hospital for Children (Siem Reap, Cambodia), Mahosot Hospital, a large national referral centre (Vientiane, Laos), and the Shoklo Malaria Research Unit, serving migrant and refugee populations on the Thailand-Myanmar border (Mae Sot, Thailand), including antibiotics tested for > 40% of the isolates across all study years, and comprising 6154 isolates, 2008–2019. Whilst these are not entirely representative of the countries, they have information on resistance in both bloodstream and other specimens, information generally not available elsewhere. Here, antimicrobial susceptibility testing was conducted using disk diffusion and results interpreted using Clinical and Laboratory Standards Institute (CLSI) 2019 breakpoints. Intermediate isolates were considered non-susceptible. Extended spectrum beta-lactamase (ESBL) status was confirmed using the double disk method (ceftazidime ± clavulanate and cefotaxime ± clavulanate) as a ≥ 5 mm increase in a zone diameter for either agent with clavulanic acid. Resistance proportions, time-series cross-correlations, extent of agreement, and meta-analyses/regression were performed as for the Oxfordshire dataset above.

We conducted all analyses using R 3.5.1, using the metafor package for meta-analyses^30^.

## Supplementary Information

Supplementary Information.

## Data Availability

ATLAS data is publicly available^14^. Data from Oxfordshire are available from the Infections in Oxfordshire Research Database, subject to an application meeting the ethical and governance requirements of the database (contact email iord@ndm.ox.ac.uk). Requests for data from individual hospitals in Laos, Cambodia and Thailand should be made to Vilada Chansamouth, Paul Turner, Clare L Ling respectively. All statistical analyses were performed using standard functions in the following R packages: ggplot2 (version 3.1.0), metafor (version 2.1-0). Code used for data analysis is available upon request from the corresponding author.
